# Oncoplastic breast surgery in older women with primary breast cancer: systematic review

**DOI:** 10.1093/bjs/znad161

**Published:** 2023-06-13

**Authors:** Zoe Chia, Rachel X N Lee, Maria J Cardoso, Kwok Leung Cheung, Ruth M Parks

**Affiliations:** Nottingham Breast Cancer Research Centre, University of Nottingham, Nottingham, UK; King’s Mill Hospital, Sherwood Forest Hospitals NHS Foundation Trust, UK; Nottingham Breast Cancer Research Centre, University of Nottingham, Nottingham, UK; Queen’s Medical Centre Campus, Nottingham University Hospitals NHS Trust, Nottingham, UK; Nottingham Breast Cancer Research Centre, University of Nottingham, Nottingham, UK; Breast Unit, Champalimaud Foundation and Faculty of Medicine, University of Lisbon, Lisbon, Portugal; Nottingham Breast Cancer Research Centre, University of Nottingham, Nottingham, UK; School of Medicine, University of Nottingham, Nottingham, UK; Nottingham Breast Cancer Research Centre, University of Nottingham, Nottingham, UK; School of Medicine, University of Nottingham, Nottingham, UK

## Abstract

**Background:**

Oncoplastic procedures allow excision of larger breast tumours, or unfavourable tumour/breast ratio lesions while achieving a good cosmetic outcome. This increases the pool of patients eligible for breast conservation over mastectomy, reducing the need for more extensive surgery in older women and potentially improving their quality of life. Nonetheless, studies to date suggest a poor uptake of oncoplastic breast surgery in the older group. This review aimed to establish whether a disparity in uptake of oncoplastic breast surgery exists between older and younger women, and to explore the underlying reasons for this.

**Methods:**

A literature search was conducted on 17 January 2022 using MEDLINE and Embase. Eligible studies comprised full-text articles of patients who underwent oncoplastic breast surgery for primary invasive breast cancer, and included those aged at least 65 years.

**Results:**

Ten published studies were identified. One study was ranked as providing level 2 evidence, and the remainder were level 3. A total of 567 women underwent oncoplastic breast surgery for primary breast cancer, of whom only 61 (10.8 per cent) were aged 65 years or older. None of the studies directly compared younger with older women, or explored the underlying factors contributing to this discrepancy in uptake.

**Conclusion:**

This review has demonstrated a lower uptake of oncoplastic breast surgery in older compared with younger women. Given the increasing number of older women living with breast cancer who may be eligible for breast-conserving surgery, further research into this area is required.

## Teaser

Studies to date suggest a poor uptake of oncoplastic breast surgery in older women. This literature review aimed to establish whether a disparity in uptake exists between older and younger women, and to identify any underlying reasons for this. The review demonstrated a lower uptake of oncoplastic breast surgery in older compared with younger women; however, the underlying contributory factors have yet to be explored.

## Introduction

Breast cancer is the most common female malignancy worldwide, and the incidence increases with age^1,2^. In 2020, the World Cancer Research Fund^1^ reported more than 2.26 million new breast cancer diagnoses among women worldwide. Given the growth of the older population^3^, now designated as ‘the silver tsunami’, combined with improvement in breast cancer survival, the proportion of older women (aged at least 65 years) living with the long-term consequences of breast cancer treatment is rising^2,4^.

Oncoplastic breast surgery (OBS) combines breast conservation and plastic surgery techniques with the aim of achieving safe oncological resection, as well as maintaining good cosmetic outcome. Several studies^5–8^ including two systematic reviews have reported on the oncological safety of OBS. The development of oncoplastic techniques in breast cancer surgery has significantly advanced the discipline over the years by minimizing breast asymmetry with the immediate reconstruction of sizeable resection defects, and not infrequently with symmetrization of the contralateral breast. This allows excision of larger tumour sizes with free resection margins, increasing the pool of patients eligible for breast conservation^9^.

The benefits of OBS are manifold, and include reducing the need for more extensive surgery with mastectomy and reconstruction, along with improvements in aesthetic and quality-of-life outcomes^10–13^. Studies^14,15^ have shown that functional status can decline after breast cancer surgery, and this is compounded by the intensity of the surgery itself. Therefore, OBS may be better than mastectomy with reconstruction in preserving functional status in older women. Despite this and the ageing population, there is a paucity of high-level evidence on the uptake of immediate reconstruction following breast-conserving surgery (BCS) in older women.

The National Institute for Health and Care Excellence Breast Cancer Quality Standard^16^ states that, irrespective of age, patients with early breast cancer should be offered the full range of appropriate management unless precluded by significant co-morbidity. This was further endorsed by the UK All Party Parliamentary Group on Breast Cancer in 2013, which stipulated that treatment choices should be determined by patient fitness and not chronological age alone^17^. Moreover, the European Society of Breast Cancer Specialists and the International Society of Geriatric Oncology^18^ recommend that patients aged 70 years or more should be offered the same surgical options as younger patients, including oncoplastic surgery, once preferences and co-morbidities have been considered.

Although not specific to older women, there is a larger body of evidence available regarding postmastectomy immediate breast reconstruction in contrast to OBS^4,19–22^. A recent systematic review by Lee *et al.*^23^ demonstrated a significant disparity in the uptake of postmastectomy immediate breast reconstruction in older (15 144 of 152 836, 10 per cent) compared with younger (142 533 of 313 298, 45.5 per cent) women, and highlighted several themes that may contribute to this, such as patient-, physician-, and system-associated factors. Therefore, it is hypothesized that a disparity in uptake similarly exists for OBS. Given the scarcity of high-level evidence on OBS in the older age group, the aims of this review were to establish whether a disparity in the uptake of OBS between older (aged at least 65 years) and younger women exists, and to identify any underlying reasons for this.

## Methods

The research question was developed using the PICO (Population, Intervention, Comparison, Outcome) framework, whereby the population included older women after OBS, the intervention was immediate reconstruction with OBS, the comparison was uptake of OBS in younger women, and the outcome was to assess whether a disparity in uptake of OBS between older and younger women exists. The review was conducted in adherence with the PRISMA statement^24^.

### Search strategy

**Fig. 1:**
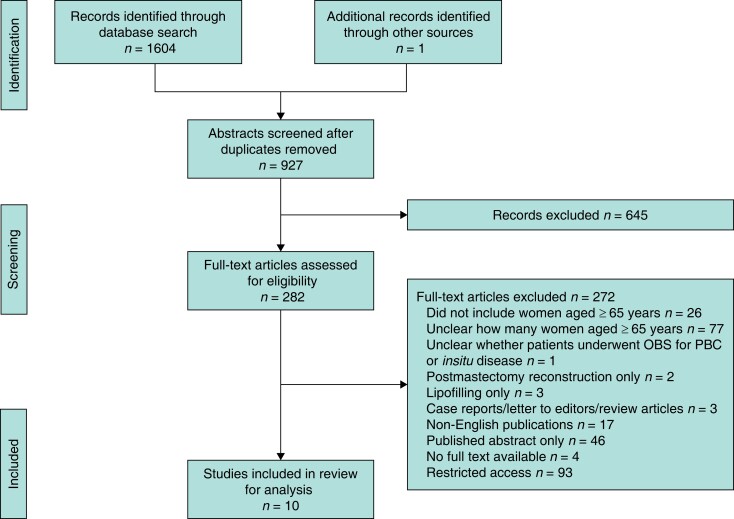
PRISMA flow chart showing selection of articles for review OBS, oncoplastic breast surgery; PBC, primary breast cancer.

A systematic search of the literature was undertaken in MEDLINE and Embase databases via Ovid using a search strategy that was finalized with a clinical librarian. The search was completed in January 2022; the study selection process is outlined in *Fig. 1*.

The cut-off age of 65 years and above was used, in line with the common definition of ‘older persons’ within medical literature^25^. A cut-off age of 70 years and above has also been used to describe a person as older^26^; however, using this definition would have limited the number of studies available for review. The framework for each search strategy, along with the search terms used for both MEDLINE and Embase databases, is presented in *Appendix S1*.

One reviewer undertook the database searches and imported all results into the Endnote^TM^ (Philadelphia, PA, USA) reference manager, subsequently removing any duplicates. Two independent reviewers were involved in the following stages. In stage 1, abstracts were screened independently to identify the subset warranting a full-text review. In stage 2, full-text articles were assessed independently for eligibility as guided by the inclusion and exclusion criteria. The final full-text articles were included based on both reviewers’ consensus, with a third reviewer available to resolve discrepancies.

The inclusion criteria were: full-text articles published in English; peer reviewed only; female participants; studies including any number of women aged at least 65 years; and studies involving patients who had undergone BCS with immediate reconstruction for primary breast cancer.

Exclusion criteria were: studies that did not fulfil the inclusion criteria; immediate breast reconstruction not discussed or unable to be differentiated from delayed breast reconstruction; patients with ductal carcinoma *in situ*; prophylactic surgery; studies carried out in participants who had undergone any type of mastectomy; studies in which the number of women aged 65 years or more who had undergone OBS was unclear; review articles, editorials, case reports, and letters to editors; and articles with restricted access.

### Data extraction

Data extracted by the primary reviewer included: country and year of publication; study aim; total number of patients (older and younger groups); number of patients who underwent OBS for primary breast cancer (older and younger groups); type of OBS; factors affecting uptake of OBS; and study conclusion.

### Critical appraisal

The Harbour and Miller system^27^ was used to assess the studies included and their level of evidence. Risk of bias was evaluated using the Cochrane risk-of-bias assessment tool^28^ at both study and outcome level.

## Results

In total, 10 studies fulfilled the inclusion criteria and were analysed in this review. These studies were published between 2004 and 2017; they included a total of 567 women, 61 of whom were older, and underwent immediate reconstruction following BCS for primary breast cancer.

### General characteristics

Characteristics of the 10 included studies are detailed in *Table S1*. Six studies were conducted in Europe and four in East Asia. Nine of the studies included were case series (more than 3 patients) and one was a cohort study. Six studies were prospective in design and three were retrospective; this was unclear for the remaining study. One study^29^ was rated as having level 2 evidence, whereas the remaining nine studies^30–38^ presented level 3 evidence^27^. A summary of the risk-of-bias assessment for the studies included in this review is provided in *Table S2*.

The findings are presented in line with the objectives of this review. First the disparity in uptake of OBS between older and younger women is compared, and then the possible underlying factors contributing to this disparity are explored.

### Disparity in uptake of OBS in older and younger women

The 10 studies included a total of 567 women who underwent OBS for primary breast cancer. Older women accounted for 10.8 per cent (61 of 567), compared with 89.2 per cent (506 of 567) for younger women. None of the studies reviewed specifically explored the underlying factors contributing to this disparity in uptake. The feasibility and safety of various oncoplastic techniques were, however, described and are examined below.

### Evaluation of safety of OBS

The oncological safety of OBS in primary breast cancer was evaluated by three studies in this review. A 2016 study by De Lorenzi *et al.*^29^ compared a large series of 454 patients with breast cancer who underwent OBS with a control group of 908 patients who had breast conservation alone. In the OBS group, only 6.4 per cent (29 of 454) were older women. Both groups were matched for age, year of surgery, tumour size, and systemic treatment protocol. Prognostic and biological features were also balanced. The overall survival rate at 10 years was 91.4 per cent in the OBS group and 91.3 per cent in the control group. Disease-free survival at 10 years was lower in the OBS group (69 per cent *versus* 73.1 per cent in the control group), but this difference was not statistically significant. The results of this study were reported to include the entire patient cohort, so no data specific to older women were obtained.

A case series by Woerdeman *et al.*^30^ examined the long-term local control and cosmetic outcome of patients managed with preoperative radiotherapy and immediate reconstruction with the latissimus dorsi myocutaneous flap (LDMCF) in T2 and T3 breast cancers. Only 1 in 20 women in this study were aged 65 years and above. A local control rate of 95 per cent owing to an observed locoregional recurrence in one patient who declined adjuvant chemotherapy was noted. The 5-year survival and 10-year actuarial survival rates were reported as 75 and 60 per cent respectively. Overall, the cosmetic outcome was noted to be satisfactory. Data reported for local control and cosmetic outcome were not exclusive to older women.

In 2008, the case series by De Lorenzi *et al.*^31^ examined 63 patients aged over 65 years who underwent immediate reconstruction after breast cancer surgery, of whom 14 underwent OBS. Adjuvant radiotherapy was delivered to 14 patients (22.2 per cent), chemotherapy to 21 (33.3 per cent), and endocrine therapy to 39 (61.9 per cent). Additionally, neoadjuvant chemotherapy and preoperative radiotherapy were delivered to two (3.2 per cent) and seven patients (11.1 per cent) respectively. One of 14 patients from the OBS cohort experienced a postoperative wound infection. Three patients were lost to follow-up; of the remaining cohort, 84 per cent were noted to be disease-free, 6.3 per cent presented with local recurrence, 7.9 per cent with systemic disease, and 1.6 per cent with both local and metastatic disease.

### Evaluation of feasibility of oncoplastic techniques

Seven studies^32–38^ in this review discussed the feasibility of several oncoplastic techniques in the management of primary breast cancer.

#### Reconstruction of central defects and nipple–areolar complex

Cartensen *et al.*^32^ explored the use of reduction techniques and the anterior intercostal artery perforator (AICAP) flap for central breast cancers involving the nipple–areolar complex. Sixteen of 20 patients underwent OBS for primary breast cancer, of whom seven were older women. After surgery, four patients were reoperated because of involved margins, two of whom were in older women. Three patients experienced wound infections, two of whom were older women. Overall, the majority were satisfied with the cosmetic outcome. In this study, it was possible to obtain data specific to older women for postoperative complications and re-excision rate only.

Likewise, Schoeller *et al.*^33^ described a technique for reconstructing the nipple–areolar complex following central tumour excision. This study included nine patients, six of whom had invasive breast cancer; three of these were older women. The operative principles used here resembled those of breast reduction surgery, and the neonipple and areola were reconstructed using a local skin flap and full-thickness skin graft. The neonipple healed well in all patients and the skin graft took successfully in eight patients. Overall, the aesthetic results were reported as good to excellent. Data specific to older women for postoperative healing were provided in this study.

#### Autologous and perforator flaps

Carrasco-Lopez *et al.*^34^ described the anatomy of AICAP flaps from cadaveric dissection and reviewed their technique in using these flaps for OBS. The clinical study included 14 patients, two of whom were older women. Generally, clinical outcomes were good, with only one case of haematoma formation and partial flap failure. Mean BREAST-Q scores were compared before and after surgery, and the only domain that demonstrated a significant drop was physical well-being, which was thought likely to be secondary to changes in skin quality and arm mobility. In this study, it was possible to obtain data specific to older women.

Izumi *et al.*^35^ reported the use of medial circumflex femoral artery perforator flaps in immediate reconstruction after BCS in 15 patients, two of whom were older women. For recipient vessel anastomosis of the harvested flap, internal mammary branches were used in medial defects, and serratus or thoracodorsal branches for lateral defects. Complications included partial flap necrosis in two patients and wound dehiscence in one. Cosmetic outcomes were reported as excellent for eight patients, very good for four, good for two, and fair for one. Data specific to older women were provided in this study.

Lee *et al.*^36^ investigated the use of the combined LDMCF, thoracoepigastric flap, and inferior-pedicled rotational local flap for immediate reconstruction of large resection defects in ptotic breasts. Eighteen patients underwent reconstruction for invasive cancer, one of whom was an older woman. Postoperative complications included wound dehiscence. Cosmetic outcomes were mostly reported as fair to excellent. In this study, data specific to older women were provided, but postoperative complications were reported for the entire study cohort.

#### Free dermal fat grafts

Two studies by Kijima *et al.*^37,38^ described the use of free dermal fat grafts (FDFGs) in OBS. In 2007, six patients underwent surgery for primary breast cancer; only one was an older woman. Here, the FDFG was harvested via a low abdominal transverse incision. Following surgery, no positive margins were identified. Subsequently in 2013, the technique was modified to harvest the FDFG from the lateral abdomen via a vertical incision instead, where the scar was reported to be shorter. This series consisted of four patients, one of whom was an older woman. Good postoperative symmetry was observed in both studies. Some data specific to older women were provided in both studies, but cosmetic outcome was reported for the entire patient group.

## Discussion

The main limitation of this review is the small number of articles included. Moreover, none presented a direct comparison of the uptake of OBS between older and younger women. Most of the studies included provided level 3 evidence, whereas one retrospective cohort study was rated as having level 2 evidence. Although there was a lack of studies with high-level evidence, given that the aim was to identify any disparity in the uptake of OBS in older *versus* younger women, RCTs may not represent the most suitable assessment method. Additionally, the underlying factors that contributed to the discrepancy in uptake were not explored. A potential confounding factor is that not all studies provided data specific to older women. Furthermore, taken that this review comprised only studies in which women aged at least 65 years were involved, it does not provide a true representation of the real-life disparity in uptake of OBS, which is likely to be more significant.

As far as the authors are aware, this is the first systematic review to specifically quantify the disparity in uptake of OBS between older and younger women. A separate literature review by Lee *et al.*^23^ was conducted with similar objectives, but with a focus on postmastectomy immediate breast reconstruction instead. The present review reported an uptake of OBS of only 10.8 per cent (61 of 567) in older women *versus* 89.2 per cent (506 of 567) among younger women.

Several guidelines^16–18^ have stated that patients should be offered the same breast cancer treatment options irrespective of age, but with careful consideration of individual preferences and co-morbidities. Bowman *et al.*^39^ surveyed 75 older patients and their attitudes toward breast reconstruction. Over 90 per cent of patients felt that age should not be a factor in considering candidacy for breast reconstruction. Another study^40^ stated that reconstruction was rarely discussed with older women, although all would have liked to have the option. Although these studies focused primarily on patients following a mastectomy, it is possible that the outlook of older women with OBS may be similar.

None of the studies appraised in this review investigated the underlying factors resulting in a disparity in uptake of OBS between older and younger women. Therefore, the contributory factors identified in the literature review by Lee *et al.*^23^, which similarly reported a lower uptake of postmastectomy immediate breast reconstruction in older (10 per cent, 15 144 of 152 836) compared with younger women (45 per cent, 142 533 of 313 298), are discussed below. The three main themes identified included physician-, system- and patient-associated factors.

For physician-associated factors, it was noted that older women are highly dependent on surgeons’ recommendations when contemplating immediate reconstruction after mastectomy^23^, preferring to obtain treatment information directly from healthcare professionals^40^. Nonetheless, research^40–42^ has shown that clinicians tend to provide more detailed information and support to younger patients. Therefore, it is important that awareness is raised to allow clinicians to recognize these potential biases and work towards overcoming it.

Next, system-associated factors include the geographical inequalities that arise from OBS services being centralized to specialist centres^43^. Studies^44–46^ have demonstrated that operations performed in larger academic or cancer centres with higher volume are associated with an increased rate of immediate reconstruction after breast cancer surgery. This may deter older patients from seeking out specialist treatment in tertiary hospitals owing to the need for distant travel.

In addition, patient-associated factors, such as older age, co-morbidities, and ethnic cultural values, may affect the choice to undergo immediate reconstruction. It was noted that African American, Hispanic, and Asian women were less likely to undergo postmastectomy immediate breast reconstruction as these communities do not consider reconstruction as a necessary surgical procedure^23,47^. Further research into this area is required to precisely identify factors contributing towards the disparity in uptake of OBS, and subsequently progress towards narrowing the gap.

The findings of this review support the notion that OBS is a reliable treatment option for the management of primary breast cancer. Only the study by De Lorenzi *et al.*^31^, however, was conclusive for older women, deducing that advanced age itself should not be a contraindication to immediate breast reconstruction, as it is safe in well selected patients.

Immediate oncoplastic reconstruction has the added benefit of allowing wider excisions of breast tissue, providing an oncological advantage by reducing the likelihood of positive resection margins and the need for re-excision. A narrative review of reconstructive breast surgery in older patients by James *et al.*^13^ examined several studies, and reported a lower re-excision rate in patients who underwent therapeutic mammoplasty in comparison to breast conservation alone. This is important for older women as it reduces the need for a second operation with associated anaesthetic and surgical risks.

Generally, OBS was well tolerated despite age, with no unfavourable postoperative sequelae delaying adjuvant radiotherapy^31^. Given that older women have coexisting health and social care needs, a comprehensive geriatric assessment (CGA) should be used to allow clinicians to establish patients’ baseline functional status, physiological age, and individual care needs^18^. The CGA has been documented as a useful adjunct in older women with early breast cancer, helping to determine patient suitability for treatment planning^48^. The National Audit of Breast Cancer in Older Patients 2022 annual report^49^ similarly recommends the use of a fitness assessment form to evaluate objectively the health of older patients and minimize their risk of undertreatment or overtreatment.

The studies in this review described numerous different OBS procedures with overall complication rates of between 14 and 25 per cent. There was, however, no conclusive evidence on its feasibility in older women, possibly owing to its underuse in this age group. Two studies^50,51^ have reported higher complication rates and poorer physical outcomes following implant-based *versus* autologous reconstruction. As a result, implant reconstruction may be considered less appropriate for older women given the potential need for reoperation for complications. Additionally, OBS may be more suitable in older women with ptotic breasts, as therapeutic mammoplasties can deliver better cosmetic results with bilateral symmetrization^13,22^.

Given the array of oncoplastic techniques available, decision-making can be overwhelming when associated with a new cancer diagnosis. A study of 563 older women with early breast cancer described how receiving treatment in keeping with personal preferences about body image is important for mental health^52^. Therefore, interventions should be made available to support older women in their decision-making process, such as preconsultation educational groups, printed/online decision aids, and an interactive digital system^53^.

A disparity exists in uptake of OBS among women of different ages. This review has highlighted how the factors contributing to this disparity have yet to be explored, representing an important area for research to inform future clinical practice. It is hypothesized that there are two categories of factors that may be contributing to a discrepancy in uptake of OBS in older women: non-modifiable factors, such as patient preference and co-morbidities, as well as modifiable factors. The latter include clinician bias, poor access to tertiary OBS services, and deficient patient information and support surrounding decision-making. Future strategies could include the development of decision support tools, along with the need for patient-reported outcomes to highlight areas for future development. In line with this, the Getting It Right First Time National Specialty Report for Breast Surgery^54^ in the UK recommends that oncoplastic multidisciplinary teams be established in all hospitals to provide support and access to oncoplastic conservation unrestricted by resources.

## Supplementary Material

znad161_Supplementary_Data

## Data Availability

Data sharing is not applicable to this article, as no data sets were generated or analysed during this study.
