# Supplementary material for: Oncoplastic breast surgery in older women with primary breast cancer: systematic review
Source: Br J Surg. 2023 Jun 13;110(10):1309–15. doi: 10.1093/bjs/znad161 (PMC10480033; doi:10.1093/bjs/znad161)
Supplement: znad161_Supplementary_Data [file znad161_supplementary_data.docx]

**Title**

Oncoplastic Breast Surgery in Older Women with Primary Breast Cancer: Systematic Review

**Authors**

Zoe Chia^1,2^, Rachel XN Lee^1,3^, Maria J Cardoso^1,5^, Kwok Leung Cheung^1,4^, Ruth M Parks^1,4^

^1^Nottingham Breast Cancer Research Centre, University of Nottingham, UK

^2^King’s Mill Hospital, Sherwood Forest Hospitals NHS Foundation Trust

^3^Queen’s Medical Centre Campus, Nottingham University Hospitals NHS Trust
^4^School of Medicine, University of Nottingham, UK
^5^Breast Unit, Champalimaud Foundation and Faculty of Medicine University of Lisbon, Lisbon, Portugal

**Corresponding Author**

Ruth Mary Parks

School of Medicine,

University of Nottingham,

Royal Derby Hospital Centre,

Uttoxeter Road,

Derby, DE22 3DT, UK

E-mail: Ruth.Parks@nottingham.ac.uk

ORCID: <https://orcid.org/0000-0003-3832-7431>

Twitter: @ruthmparks

**Supplementary Materials - Index**

| **Supplementary Appendixes** |  |
| --- | --- |
| Appendix S1: Search strategy for Medline and Embase | *page 3* |
| Appendix S2: PRISMA checklist | *page 4 – 6* |
|  |  |
| **Supplementary Figures and Tables** |  |
| Table S1: Characteristics and findings of the 10 included studies | *page 8 – 11* |
| Table S2: Summary of risk of bias assessment | *page 12 – 13* |
|  |  |
| **References** | *page 14 – 17* |
|  |  |

**Appendix S1**: Search strategy for Medline and Embase


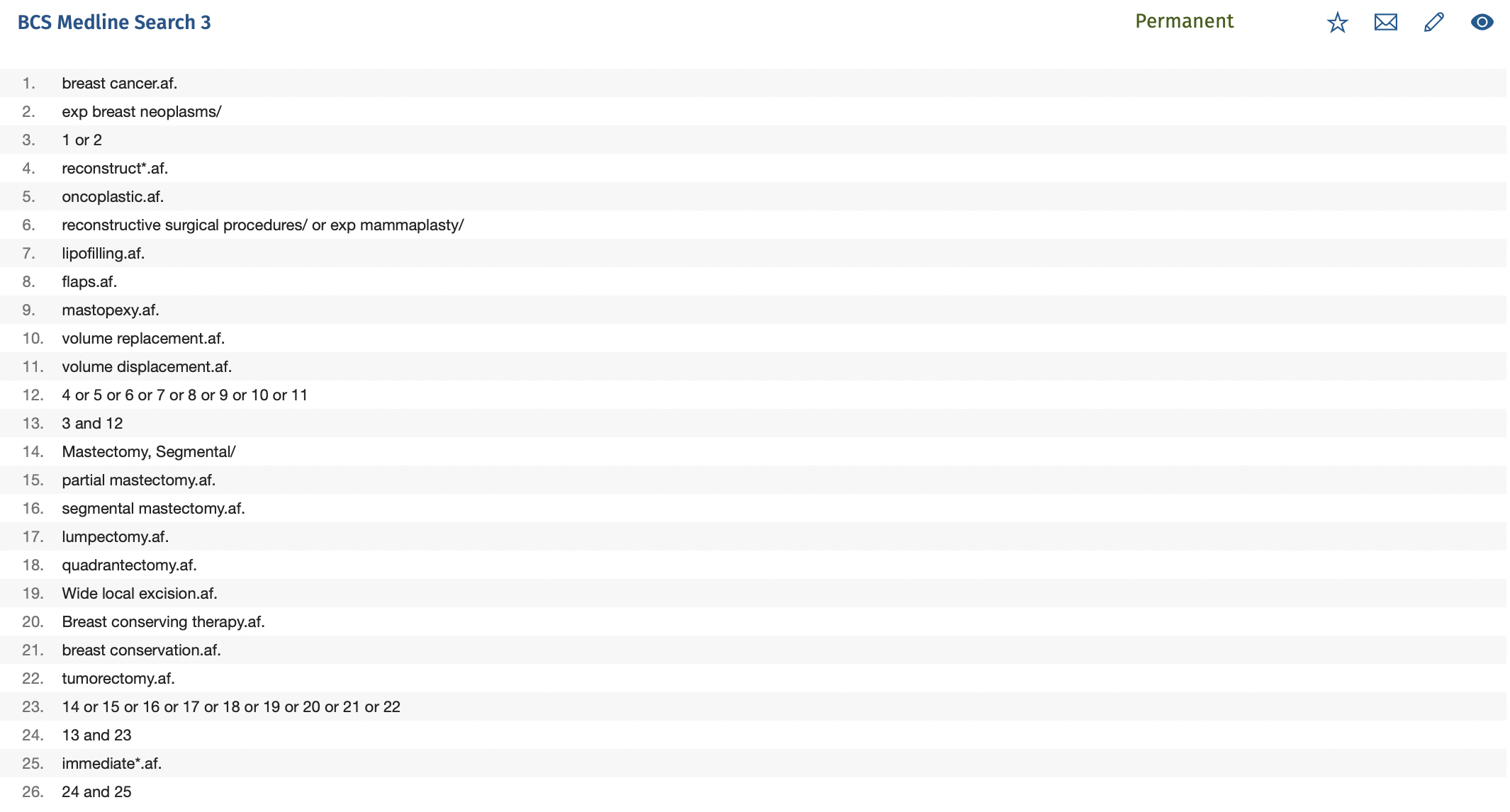


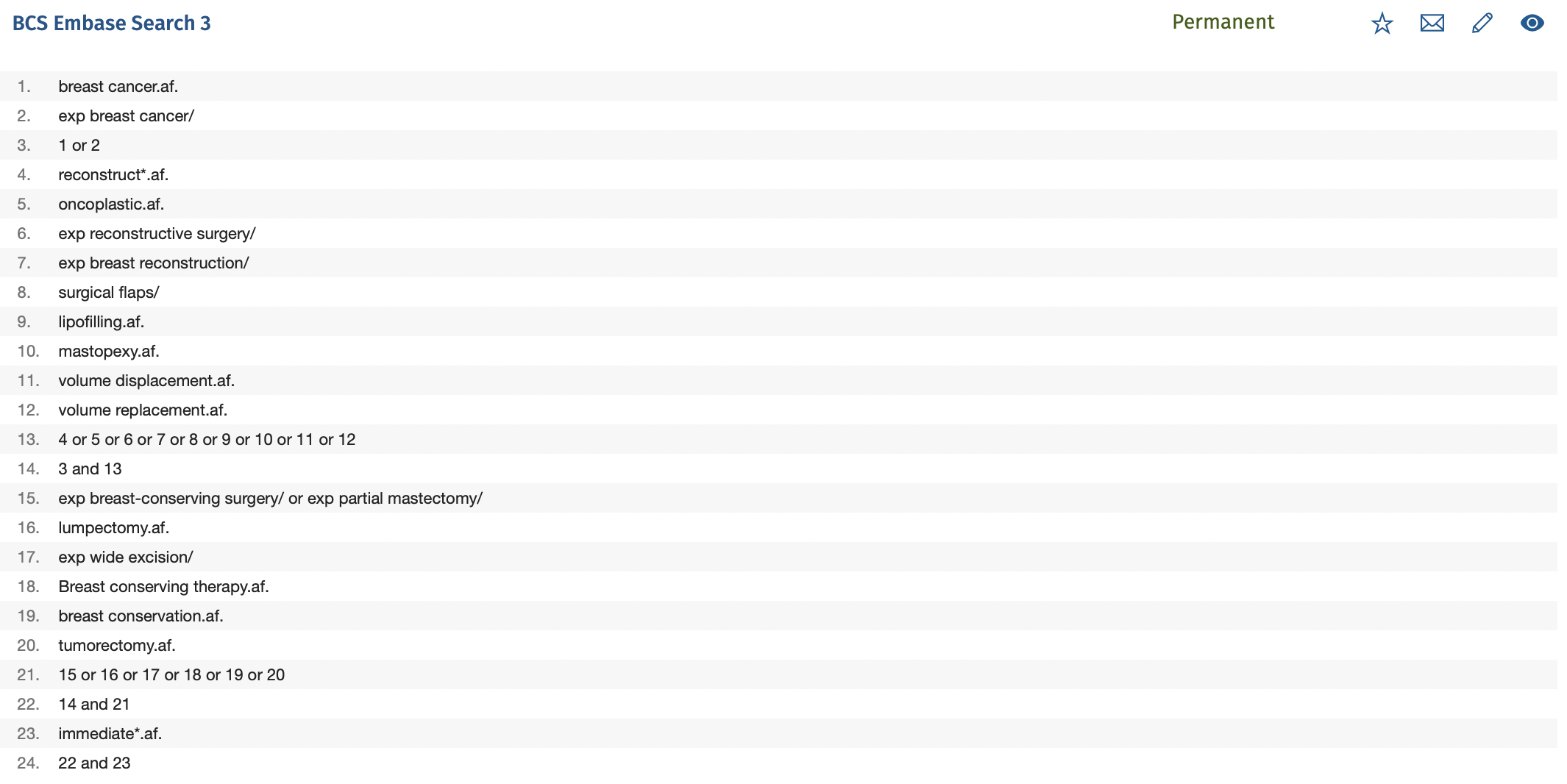


**Appendix S2**: PRISMA checklist.

| **Section and Topic** | **Item #** | **Checklist item** | **Location where item is reported** |
| --- | --- | --- | --- |
| **TITLE** | | |  |
| Title | 1 | Identify the report as a systematic review. | 1 |
| **ABSTRACT** | | |  |
| Abstract | 2 | See the PRISMA 2020 for Abstracts checklist. | 2–3 |
| **INTRODUCTION** | | |  |
| Rationale | 3 | Describe the rationale for the review in the context of existing knowledge. | 3–4 |
| Objectives | 4 | Provide an explicit statement of the objective(s) or question(s) the review addresses. | 4 |
| **METHODS** | | |  |
| Eligibility criteria | 5 | Specify the inclusion and exclusion criteria for the review and how studies were grouped for the syntheses. | 5–6 |
| Information sources | 6 | Specify all databases, registers, websites, organisations, reference lists and other sources searched or consulted to identify studies. Specify the date when each source was last searched or consulted. | 5 |
| Search strategy | 7 | Present the full search strategies for all databases, registers and websites, including any filters and limits used. | 5–6 |
| Selection process | 8 | Specify the methods used to decide whether a study met the inclusion criteria of the review, including how many reviewers screened each record and each report retrieved, whether they worked independently, and if applicable, details of automation tools used in the process. | 5–6 |
| Data collection process | 9 | Specify the methods used to collect data from reports, including how many reviewers collected data from each report, whether they worked independently, any processes for obtaining or confirming data from study investigators, and if applicable, details of automation tools used in the process. | 5–6 |
| Data items | 10a | List and define all outcomes for which data were sought. Specify whether all results that were compatible with each outcome domain in each study were sought (e.g. for all measures, time points, analyses), and if not, the methods used to decide which results to collect. | 4 |
|  | 10b | List and define all other variables for which data were sought (e.g. participant and intervention characteristics, funding sources). Describe any assumptions made about any missing or unclear information. | 5–6 |
| Study risk of bias assessment | 11 | Specify the methods used to assess risk of bias in the included studies, including details of the tool(s) used, how many reviewers assessed each study and whether they worked independently, and if applicable, details of automation tools used in the process. | 6 |
| Effect measures | 12 | Specify for each outcome the effect measure(s) (e.g. risk ratio, mean difference) used in the synthesis or presentation of results. | N/A |
| Synthesis methods | 13a | Describe the processes used to decide which studies were eligible for each synthesis (e.g. tabulating the study intervention characteristics and comparing against the planned groups for each synthesis (item #5)). | 5–6 |
|  | 13b | Describe any methods required to prepare the data for presentation or synthesis, such as handling of missing summary statistics, or data conversions. | N/A |
|  | 13c | Describe any methods used to tabulate or visually display results of individual studies and syntheses. | 33 |
|  | 13d | Describe any methods used to synthesize results and provide a rationale for the choice(s). If meta-analysis was performed, describe the model(s), method(s) to identify the presence and extent of statistical heterogeneity, and software package(s) used. | N/A |
|  | 13e | Describe any methods used to explore possible causes of heterogeneity among study results (e.g. subgroup analysis, meta-regression). | N/A |
|  | 13f | Describe any sensitivity analyses conducted to assess robustness of the synthesized results. | N/A |
| Reporting bias assessment | 14 | Describe any methods used to assess risk of bias due to missing results in a synthesis (arising from reporting biases). | 6 |
| Certainty assessment | 15 | Describe any methods used to assess certainty (or confidence) in the body of evidence for an outcome. | 6–7 |
| **RESULTS** | | |  |
| Study selection | 16a | Describe the results of the search and selection process, from the number of records identified in the search to the number of studies included in the review, ideally using a flow diagram. | 32 |
|  | 16b | Cite studies that might appear to meet the inclusion criteria, but which were excluded, and explain why they were excluded. | N/A |
| Study characteristics | 17 | Cite each included study and present its characteristics. | 33 |
| Risk of bias in studies | 18 | Present assessments of risk of bias for each included study. | 34 |
| Results of individual studies | 19 | For all outcomes, present, for each study: (a) summary statistics for each group (where appropriate) and (b) an effect estimate and its precision (e.g. confidence/credible interval), ideally using structured tables or plots. | 33 |
| Results of syntheses | 20a | For each synthesis, briefly summarise the characteristics and risk of bias among contributing studies. | 34 |
|  | 20b | Present results of all statistical syntheses conducted. If meta-analysis was done, present for each the summary estimate and its precision (e.g. confidence/credible interval) and measures of statistical heterogeneity. If comparing groups, describe the direction of the effect. | 6–12 |
|  | 20c | Present results of all investigations of possible causes of heterogeneity among study results. | N/A |
|  | 20d | Present results of all sensitivity analyses conducted to assess the robustness of the synthesized results. | N/A |
| Reporting biases | 21 | Present assessments of risk of bias due to missing results (arising from reporting biases) for each synthesis assessed. | 34 |
| Certainty of evidence | 22 | Present assessments of certainty (or confidence) in the body of evidence for each outcome assessed. | 34 |
| **DISCUSSION** | | |  |
| Discussion | 23a | Provide a general interpretation of the results in the context of other evidence. | 13–19 |
|  | 23b | Discuss any limitations of the evidence included in the review. | 21 |
|  | 23c | Discuss any limitations of the review processes used. | 21 |
|  | 23d | Discuss implications of the results for practice, policy, and future research. | 19–22 |
| **OTHER INFORMATION** | | |  |
| Registration and protocol | 24a | Provide registration information for the review, including register name and registration number, or state that the review was not registered. | Not registered |
|  | 24b | Indicate where the review protocol can be accessed, or state that a protocol was not prepared. | Please request from authors |
|  | 24c | Describe and explain any amendments to information provided at registration or in the protocol. | Please request from authors |
| Support | 25 | Describe sources of financial or non-financial support for the review, and the role of the funders or sponsors in the review. | 22 |
| Competing interests | 26 | Declare any competing interests of review authors. | 22 |
| Availability of data, code and other materials | 27 | Report which of the following are publicly available and where they can be found: template data collection forms; data extracted from included studies; data used for all analyses; analytic code; any other materials used in the review. | Please request from authors |

**Table S1:** Characteristics and findings of the 10 included studies.

| **First author** | **Country of study** | **Study design** | **L** | **Aims of study** | **N** | **Total no. of women who had OBS for PBC (n=567)** | **No. of women who had OBS for PBC** | | **No. of women receiving OBS for other causes* (n=9)** | **Type of OBS** | **Mean age (years)** | **Follow-up** | **Conclusion** |
| --- | --- | --- | --- | --- | --- | --- | --- | --- | --- | --- | --- | --- | --- |
|  |  |  |  |  |  |  | **Younger**  **<65 years**  **(n=506)** | **Older**  **≥65 years**  **(n=61)** |  |  |  |  |  |
| De Lorenzi F^29^  2016 | Italy | Cohort, retro-spective | 2 | To assess the safety of oncoplastic surgery for invasive primary breast cancer. | 1362 | 454 | 425 | 29 | 0 | Flaps, mastopexy, mammoplasty, implants | Unclear | Median of 7.2 years | OBS is a safe and reliable treatment option for the management of invasive breast cancer. |
| Woerdeman L. A. E^30^  2004 | Nether-lands | Case series,  pro-spective | 3 | To evaluate long-term local oncologic control and cosmetic outcome of preoperative radiotherapy and IBR with the LDMCF in patients with large T2 and T3 breast cancers. | 20 | 20 | 19 | 1 | 0 | LDMCF | 49  (29-65) | Minimum of 5 years | Preoperative radiotherapy and immediate LDMCF reconstruction after WLE is an oncologically safe and cosmetically rewarding, but logistically straining treatment option for patients with large T2 and T3 breast cancers. |
| De Lorenzi F^31^  2008 | Italy | Case series,  retro-spective | 3 | To demonstrate that breast reconstruction is feasible and safe in the older cohort. | 63 | 14 | 0 | 14 | 0 | Mastopexy, mammoplasty | 70  (66-81) | Mean of 43.1 months  (7.5-94.6) | Advanced age alone is not a contraindication to breast reconstruction as it can be successfully performed in well-selected patients. |
| Cartensen L^32^  2015 | Denmark | Case series, pro-spective | 3 | To suggest a guideline for the selection of an optimal surgical technique for central tumours and NAC reconstruction in small- to medium-sized breasts. | 20 | 16 | 9 | 7 | 4 | Reduction technique, AICAP | 59.9  (40-78) | Mean of 19 months (8-32) | Immediate reconstruction of the NAC with good cosmetic outcome is possible regardless of the size and shape of the breast. The AICAP flap is a good alternative in cases where therapeutic mastopexy is not feasible. |
| Schoeller T^33^  2006 | Austria | Case series,  pro-spective | 3 | To present a simple and effective technique for immediate NAC reconstruction following a central lumpectomy. | 9 | 6 | 3 | 3 | 3 | Local tissue flap with dermo-glandular pedicle, contralateral mammoplasty | 59.3  (37-74) | Mean of 18.2 months  (2-61) | The presented technique is easily achievable and expands the options of oncoplastic surgery to meet central defects with loss of the NAC. |
| Carrasco-Lopez C^34^  2017 | Spain | Case series, pro-spective | 3 | To describe the anatomy and characteristics of AICAP perforators and review surgical technique in IBR. | 14 | 14 | 12 | 2 | 0 | AICAP | 53  (34-67) | Mean of 14 months (11-20) | AICAP flaps have consistent vascularization with good perforators. It is suitable for partial breast reconstruction and does not negatively impact patient satisfaction. |
| Izumi K^35^  2013 | Japan | Case series,  pro-spective | 3 | To describe the results of IBR with the MCFAP flap after BCS. | 15 | 15 | 13 | 2 | 0 | MCFAP | 48.4  (32-74) | Mean of 8.7 months (3-13) | Given its reliable vascularity and low donor-site morbidity, the MCFAP flap is good for breast reconstruction post-BCS. |
| Seungju L^36^  2014 | Republic of Korea | Case series,  retro-spective | 3 | To investigate combining the LDMCF with two local flaps for reconstruction of large defects in ptotic breasts after partial mastectomy. | 19 | 18 | 17 | 1 | 1 | LDMCF, TEF, IPRLF | 49.6  (32-69) | Unclear | The combined LDMCF, IPRLF and thoraco-epigastric pedicled flap provides good cosmesis in large/ptotic breasts following WLE. |
| Kijima Y^37^  2007 | Japan | Case series,  pro-spective | 3 | To document the use of FDFG aimed at improving cosmetic results of inner or central breast lesions. | 7 | 6 | 5 | 1 | 1 | FDFG from lower abdomen | 49.8  (37-70) | Mean of 23.3 months (15-29) | Immediate volume replacement using FDFG for inner or central breast defects can be useful for reconstruction with good cosmetic effect. |
| Kijima Y^38^  2013 | Japan | Case series | 3 | To report the results of immediate volume replacement with a modified FDFG from the lateral abdomen in patients with early breast cancer. | 4 | 4 | 3 | 1 | 0 | FDFG from lateral abdomen | 50  (42-66) | Unclear | Using a FDFG from the lateral abdomen may be superior to the original technique from the lower abdomen for cosmesis and preserving the option of delayed breast reconstruction if required. |

* Other causes included DCIS, Paget, Phyllodes, papilloma

**AICAP:** Anterior intercostal artery perforator flap, **BCS**: Breast conserving surgery, **IBR**: Immediate breast reconstruction,

**IPRLF**: Inferior pedicled rotational local flap, **FDFG**: Free dermal fat graft, **L**: Level of evidence,

**LDMCF**: Latissimus dorsi myocutaneous flap, **MCFAP:** Medial circumflex femoral artery perforator flap, **NAC**: Nipple areola complex,

**N:** Total number of patients in the study, **OBS**: Oncoplastic breast surgery, **TEF**: Thoraco-epigastric flap, **WLE:** Wide local excision

**Table S2:** Summary of risk of bias assessment.

| **No.** | **Author** | **Selection bias** | **Performance bias** | **Attrition bias** | **Detection bias** | **Reporting bias** | **Other bias** |
| --- | --- | --- | --- | --- | --- | --- | --- |
| 1 | De Lorenzi et al 2016^27^ | + | - | - | - | - | - |
| 2 | Woerdeman et al 2004^28^ | + | - | + | - | - | - |
| 3 | De Lorenzi et al 2008^26^ | + | - | + | - | - | - |
| 4 | Cartensen et al 2015^29^ | + | - | - | - | - | - |
| 5 | Schoeller et al 2006^30^ | + | - | - | - | - | - |
| 6 | Carrasco-Lopez et al 2017^31^ | + | - | - | - | - | - |
| 7 | Izumi et al 2013^32^ | + | - | - | - | - | - |
| 8 | Seungju et al 2014^33^ | + | - | + | - | - | - |
| 9 | Kijima et al 2007^34^ | + | - | - | - | - | - |
| 10 | Kijima et al 2013^35^ | + | - | - | - | - | - |

+ High risk of bias

- Low or unclear risk of bias

Selection bias: Random sequence generation and allocation concealment

Performance bias: Blinding of participants and personnel
Attrition bias: Incomplete outcome data
Detection bias: Blinding of outcome assessment

Reporting bias: Selective reporting
Other bias: Bias due to problems not covered elsewhere

**References**

1. Breast cancer statistics: World Cancer Research Fund International [Internet]; [Available from: <https://www.wcrf.org/cancer-trends/breast-cancer-statistics/>.

2. Breast cancer incidence (Invasive): Cancer Research UK [Internet]; [Available from: <https://www.cancerresearchuk.org/health-professional/cancer-statistics/statistics-by-cancer-type/breast-cancer#heading-Six>.

3. Ortman JM, Velkoff AA, Hogan H. An Aging Nation: The Older Population in the United States. Population Estimates and Projections. Current Population Reports. United States Census Bureau; 2014. p. 25-1140.

4. Walton L, Ommen K, Audisio RA. Breast reconstruction in elderly women breast cancer: a review. Cancer Treat Rev. 2011;37(5):353-7.

5. Carter SA, Lyons GR, Kuerer HM, Bassett RL, Oates S, Thompson A, et al. Operative and Oncologic Outcomes in 9861 Patients with Operable Breast Cancer: Single-Institution Analysis of Breast Conservation with Oncoplastic Reconstruction. Ann Surg Oncol. 2016;23(10):3190-8.

6. Campbell EJ, Romics L. Oncological safety and cosmetic outcomes in oncoplastic breast conservation surgery, a review of the best level of evidence literature. Breast Cancer (Dove Med Press). 2017;9:521-30.

7. Rutherford CL, Barker S, Romics L. A systematic review of oncoplastic volume replacement breast surgery: oncological safety and cosmetic outcome. Ann R Coll Surg Engl. 2022;104(1):5-17.

8. De La Cruz L, Blankenship SA, Chatterjee A, Geha R, Nocera N, Czerniecki BJ, et al. Outcomes After Oncoplastic Breast-Conserving Surgery in Breast Cancer Patients: A Systematic Literature Review. Ann Surg Oncol. 2016;23(10):3247-58.

9. Macmillan RD, McCulley SJ. Oncoplastic Breast Surgery: What, When and for Whom? Curr Breast Cancer Rep. 2016;8:112-7.

10. Kelemen P, Pukancsik D, Újhelyi M, Sávolt Á, Kovács E, Ivády G, et al. Comparison of clinicopathologic, cosmetic and quality of life outcomes in 700 oncoplastic and conventional breast-conserving surgery cases: A single-centre retrospective study. Eur J Surg Oncol. 2019;45(2):118-24.

11. Aristokleous I, Saddiq M. Quality of life after oncoplastic breast-conserving surgery: a systematic review. ANZ J Surg. 2019;89(6):639-46.

12. Ermoshchenkova MV, Zikiryahodjaev AD, Reshetov IV, Svyatoslavov DS, Sinelnikov MY. Psychological and Aesthetic Outcomes in Breast Cancer Patients. Plast Reconstr Surg Glob Open. 2021;9(7):e3679.

13. James R, McCulley SJ, Macmillan RD. Oncoplastic and reconstructive breast surgery in the elderly. Br J Surg. 2015;102(5):480-8.

14. Harrison CA, Parks RM, Cheung KL. The impact of breast cancer surgery on functional status in older women - A systematic review of the literature. Eur J Surg Oncol. 2021;47(8):1891-9.

15. Chia Z, Parks RM, Cheung KL. Does Breast Cancer Surgery Impact Functional Status and Independence in Older Patients? A Narrative Review. Oncol Ther. 2021;9(2):373-83.

16. Breast Cancer Quality Standard [QS12]: National Institute for Health and Care Excellence [Internet]; 2011 [updated 2016]. Available from: <https://www.nice.org.uk/guidance/qs12>.

17. All Party Parliamentary Group on Breast Cancer (APPGBC). Age is Just a Number. The Report of The Parliamentary Inquiry Into Older Age and Breast Cancer. APPGBC: London; 2013.

18. Biganzoli L, Battisti NML, Wildiers H, McCartney A, Colloca G, Kunkler IH, et al. Updated recommendations regarding the management of older patients with breast cancer: a joint paper from the European Society of Breast Cancer Specialists (EUSOMA) and the International Society of Geriatric Oncology (SIOG). Lancet Oncol. 2021;22(7):e327-e40.

19. Wong A, Snook K, Brennan M, Flitcroft K, Tucker M, Hiercz D, et al. Increasing breast reconstruction rates by offering more women a choice. ANZ J Surg. 2014;84(1-2):31-6.

20. Alderman AK, McMahon L, Jr., Wilkins EG. The national utilization of immediate and early delayed breast reconstruction and the effect of sociodemographic factors. Plast Reconstr Surg. 2003;111(2):695-703; discussion 4-5.

21. Howard-McNatt M, Forsberg C, Levine EA, DeFranzo A, Marks M, David L. Breast cancer reconstruction in the elderly. Am Surg. 2011;77(12):1640-3.

22. Maruccia M, Mazzocchi M, Dessy LA, Onesti MG. One-stage breast reconstruction techniques in elderly patients to preserve quality of life. Eur Rev Med Pharmacol Sci. 2016;20(24):5058-66.

23. Lee RXN, Cardoso MJ, Cheung KL, Parks RM. Immediate breast reconstruction uptake in older women with primary breast cancer: systematic review. Br J Surg. 2022.

24. Moher D, Shamseer L, Clarke M, Ghersi D, Liberati A, Petticrew M, et al. Preferred reporting items for systematic review and meta-analysis protocols (PRISMA-P) 2015 statement. Systematic Reviews. 2015;4(1):1.

25. Sabharwal S, Wilson H, Reilly P, Gupte CM. Heterogeneity of the definition of elderly age in current orthopaedic research. Springerplus. 2015;4:516-.

26. Jacobs JM, Maaravi Y, Cohen A, Bursztyn M, Ein-Mor E, Stessman J. Changing profile of health and function from age 70 to 85 years. Gerontology. 2012;58(4):313-21.

27. Harbour R, Miller J. A new system for grading recommendations in evidence based guidelines. Bmj. 2001;323(7308):334-6.

28. Higgins JP, Altman DG, Gøtzsche PC, Jüni P, Moher D, Oxman AD, et al. The Cochrane Collaboration's tool for assessing risk of bias in randomised trials. Bmj. 2011;343:d5928.

29. De Lorenzi F, Hubner G, Rotmensz N, Bagnardi V, Loschi P, Maisonneuve P, et al. Oncological results of oncoplastic breast-conserving surgery: Long term follow-up of a large series at a single institution: A matched-cohort analysis. Eur J Surg Oncol. 2016;42(1):71-7.

30. Woerdeman LAE, Hage JJ, Thio EA, Zoetmulder FAN, Rutgers EJT. Breast-conserving therapy in patients with a relatively large (T2 or T3) breast cancer: Long-term local control and cosmetic outcome of a feasibility study. Plastic and Reconstructive Surgery. 2004;113(6):1607-16.

31. De Lorenzi F, Rietjens M, Soresina M, Rossetto F, Bosco R, Vento AR, et al. Immediate breast reconstruction in the elderly: can it be considered an integral step of breast cancer treatment? The experience of the European Institute of Oncology, Milan. J Plast Reconstr Aesthet Surg. 2010;63(3):511-5.

32. Carstensen L, Bigaard J. Management of central breast tumours with immediate reconstruction of the nipple-areola complex; a suggested guide. Breast. 2015;24(1):38-45.

33. Schoeller T, Huemer GM. Immediate reconstruction of the nipple/areola complex in oncoplastic surgery after central quadrantectomy. Ann Plast Surg. 2006;57(6):611-5.

34. Carrasco-Lopez C, Julian Ibanez JF, Vila J, Luna Tomas MA, Navines Lopez J, Pascual Miguel I, et al. Anterior intercostal artery perforator flap in immediate breast reconstruction: Anatomical study and clinical application. Microsurgery. 2017;37(6):603-10.

35. Izumi K, Fujikawa M, Tashima H, Saito T, Sotsuka Y, Tomita K, et al. Immediate reconstruction using free medial circumflex femoral artery perforator flaps after breast-conserving surgery. J Plast Reconstr Aesthet Surg. 2013;66(11):1528-33.

36. Lee S, Lee J, Lee S, Bae Y. Oncoplastic breast surgery with latissimus dorsi myocutaneous flap for large defect in patients with ptotic breasts: is it feasible when combined with local flaps? World J Surg Oncol. 2014;12:65.

37. Kijima Y, Yoshinaka H, Owaki T, Aikou T. Early experience of immediate reconstruction using autologous free dermal fat graft after breast conservational surgery. J Plast Reconstr Aesthet Surg. 2007;60(5):495-502.

38. Kijima Y, Yoshinaka H, Hirata M, Nakajo A, Arima H, Ishigami S, et al. Immediate volume replacement using a modified free dermal fat graft from the lateral abdomen for a patient with early breast cancer. International Cancer Conference Journal. 2013;2(2):101-6.

39. Bowman CC, Lennox PA, Clugston PA, Courtemanche DJ. Breast reconstruction in older women: should age be an exclusion criterion? Plast Reconstr Surg. 2006;118(1):16-22.

40. Fenlon D, Frankland J, Foster CL, Brooks C, Coleman P, Payne S, et al. Living into old age with the consequences of breast cancer. Eur J Oncol Nurs. 2013;17(3):311-6.

41. Crooks DL. Older women with breast cancer: new understandings through grounded theory research. Health Care Women Int. 2001;22(1-2):99-114.

42. Liang W, Burnett CB, Rowland JH, Meropol NJ, Eggert L, Hwang YT, et al. Communication between physicians and older women with localized breast cancer: implications for treatment and patient satisfaction. J Clin Oncol. 2002;20(4):1008-16.

43. Hanson SE. Barriers to immediate breast reconstruction in the Canadian Universal Health Care System: Zhong T, Fernandes KA, Saskin R, et al (Univ Health Network, Toronto, Ontario, Canada; Inst for Clinical Evaluative Sciences, Toronto, Ontario, Canada; Et al) J Clin Oncol 32:2133-2141, 2014. Breast Diseases. 2015;26(2):152-3.

44. Yang B, Ren G, Song E, Pan D, Zhang J, Wang Y, et al. Current Status and Factors Influencing Surgical Options for Breast Cancer in China: A Nationwide Cross-Sectional Survey of 110 Hospitals. Oncologist. 2020;25(10):e1473-e80.

45. Gibreel WO, Day CN, Hoskin TL, Boughey JC, Habermann EB, Hieken TJ. Mastectomy and Immediate Breast Reconstruction for Cancer in the Elderly: A National Cancer Data Base Study. J Am Coll Surg. 2017;224(5):895-905.

46. Hershman DL, Richards CA, Kalinsky K, Wilde ET, Lu YS, Ascherman JA, et al. Influence of health insurance, hospital factors and physician volume on receipt of immediate post-mastectomy reconstruction in women with invasive and non-invasive breast cancer. Breast Cancer Res Treat. 2012;136(2):535-45.

47. Fu R, Chang MM, Chen M, Rohde CH. A Qualitative Study of Breast Reconstruction Decision-Making among Asian Immigrant Women Living in the United States. Plast Reconstr Surg. 2017;139(2):360e-8e.

48. Parks RM, Lakshmanan R, Winterbottom L, Al Morgan D, Cox K, Cheung K-L. Comprehensive geriatric assessment for older women with early breast cancer – a systematic review of literature. World J Surg Oncol. 2012;10(1):88.

49. Gannon M, Miller K, Medina J, Cromwell D. National Audit of Breast Cancer in Older Patients - 2022 Annual Report [Internet] England: National Audit of Breast Cancer in Older Patients; 2022 [Available from: <https://www.nabcop.org.uk/content/uploads/2022/05/NABCOP-2022-Annual-Report-V1.pdf>.

50. Lipa JE, Youssef AA, Kuerer HM, Robb GL, Chang DW. Breast reconstruction in older women: advantages of autogenous tissue. Plast Reconstr Surg. 2003;111(3):1110-21.

51. Girotto JA, Schreiber J, Nahabedian MY. Breast reconstruction in the elderly: preserving excellent quality of life. Ann Plast Surg. 2003;50(6):572-8.

52. Figueiredo MI, Cullen J, Hwang YT, Rowland JH, Mandelblatt JS. Breast cancer treatment in older women: does getting what you want improve your long-term body image and mental health? J Clin Oncol. 2004;22(19):4002-9.

53. Paraskeva N, Guest E, Lewis-Smith H, Harcourt D. Assessing the effectiveness of interventions to support patient decision making about breast reconstruction: A systematic review. The Breast. 2018;40:97-105.

54. MacNeill F, Irvine T. Breast Surgery: Getting It Right First time (GIRFT) Programme National Specialty Report [Internet] England: NHS England; 2021 [Available from: <https://associationofbreastsurgery.org.uk/media/374565/girft-report-2021.pdf>.
